# Mediodorsal thalamus and ventral pallidum contribute to subcortical regulation of the default mode network

**DOI:** 10.1038/s42003-024-06531-9

**Published:** 2024-07-23

**Authors:** Yilei Zhao, Tobias Kirschenhofer, Michael Harvey, Gregor Rainer

**Affiliations:** Section of Medicine, Faculty of Science and Medicine, University of Fribourg, Fribourg, Switzerland

**Keywords:** Neural circuits, Cognitive control

## Abstract

Humans and other animals readily transition from externally to internally focused attention, and these transitions are accompanied by activation of the default mode network (DMN). The DMN was considered a cortical network, yet recent evidence suggests subcortical structures are also involved. We investigated the role of ventral pallidum (VP) and mediodorsal thalamus (MD) in DMN regulation in tree shrew, a close relative of primates. Electrophysiology and deep learning-based classification of behavioral states revealed gamma oscillations in VP and MD coordinated with gamma in anterior cingulate (AC) cortex during DMN states. Cross-frequency coupling between gamma and delta oscillations was higher during DMN than other behaviors, underscoring the engagement of MD, VP and AC. Our findings highlight the importance of VP and MD in DMN regulation, extend homologies in DMN regulation among mammals, and underline the importance of thalamus and basal forebrain to the regulation of DMN.

## Abstract (web summary)

A study using electrophysiology, behavioral methods and modelling demonstrates the importance of the ventral pallidum and mediodorsal thalamus in DMN regulation in tree shrews.

## Introduction

The default mode network (DMN) is a collection of brain areas that tend to activate and deactivate together and exhibit enhanced functional connectivity^1,2^. The DMN is active in humans during periods of inward mental focus associated with disengagement from the external environment^3^, and encompasses the brain regions that tend to be farthest from the sensory-motor periphery^4,5^. The DMN, which is often assessed using resting-state fMRI in human subjects, is receiving increasing attention in recent years, as aberrant DMN activity appears to play an important role in a considerable number of brain disorders^6–8^. Following the discovery in humans, the DMN has also been described using functional neuroimaging in several animal species, notably including rodents and non-human primates^9–12^. Although there is some variation in the DMN between species^13^, central features of the DMN appear to be largely conserved across a variety of mammals. While the vast majority of studies related to the DMN employed functional neuroimaging, electrode recordings have documented the neural circuit activation patterns underlying the DMN in both humans and animals^14,15^ and revealed neural activity modulations in the DMN at the time of switching into a DMN brain state^16,17^. Intracranial recordings are thought to be particularly useful for identifying how specific neural circuits trigger and maintain a DMN-dominated brain state^18^.

While the DMN was initially conceptualised as a purely cortical network, recent results have highlighted that several subcortical structures, including the basal forebrain (BF), are also part of the DMN. In rats, endogenous gamma-band oscillatory activity in the ventral pallidum (VP), a nucleus of the BF, is strongly enhanced during quiet, self-directed behavioural states and suppressed during exploratory behaviours^19,20^, while in humans functional imaging studies have delineated the BF as one of the major subcortical DMN nodes^21–25^. Along similar lines, deactivation of the anterior cingulate (AC) cortex, a major cortical DMN node, leads to widespread activity suppression within other DMN nodes, including BF, as well as triggering attentional behaviours such as exploration and rearing^26^. Similarly, in rats, VP activation locks the animals in a DMN state, with profound consequences for sensory learning, while VP inactivation facilitates transitions to states requiring external focus^27^. Furthermore, task-related suppression was seen in multiple cortical DMN structures in rat intracranial recordings during operant visually based behaviour^28^. In addition to the BF, the mediodorsal (MD) thalamus, and more generally the limbic thalamus, has also emerged as another important subcortical DMN node^2,24^. MD thalamus is strongly interconnected with medial prefrontal cortical (mPFC) areas associated with the DMN, with these connectivity patterns being highly conserved across mammalian species including in humans^29–31^, and projections in tree shrews are also consistent with the other species^32,33^. Furthermore, MD thalamus may serve as a functional relay between VP and mPFC^34^, and electrical stimulation of VP modulates MD neural activity^34–36^. The VP to MD projection has also been implicated in a variety of higher cognitive functions^37,38^, regulating aspects of learning and attentional influence on task performance consistent with an involvement of the DMN. Emerging evidence thus indicates that the DMN encompasses not only cortical brain areas, but also thalamic and neuromodulatory brain structures. This study aims to study how these distinct components cooperate in DMN regulation. Our main hypotheses based on the literature were that VP nucleus would be activated in DMN-related behavioural states in the tree shrew, as it is in other mammals studied to date including humans, and that MD nucleus is modulated in a coherent manner with VP, acting as a hub that regulates DMN cortical sites in concert with VP.

The most widely used experimental approaches for DMN investigations are acquiring brain activations during the so-called resting-state^39^. Along similar lines, it has been shown that the DMN is generally activated in rat and chimpanzee during periods composed largely of quiet wakefulness in their home cage^12,19^. However, when examining animal behaviour during longer time periods inside the home cage, animals tend to spontaneously cycle through various behavioural states, offering another avenue for studying neural mechanisms of DMN regulation. Here we therefore aimed to study DMN regulation in the tree shrew (*T. belangeri*) during multiple, ~6 h duration time periods, when animals were in their home cage environment (HCE), and where animals spontaneously exhibit a variety of DMN and non-DMN associated behaviours. Although the DMN has not been previously characterised in tree shrews, we selected them as an experimental model due to their similarities to primates^40^, for example, in terms of basal forebrain neuromodulation of sensory neural circuits, as well as wake-sleep regulation^41–44^. In this context, it is necessary to determine the behaviour that tree shrews are engaged in over time, and it is not desirable to use manual scoring by human observers for this purpose, given the large amounts of video material. Fortunately, recently developed markerless pose estimation algorithms based on a deep learning architecture have become available^45,46^. These algorithms can be used to estimate the position of the nose and other body parts following manual labelling of a restricted set of video frames. This is achieved by training a convolutional neural network, e.g. a residual network with 50 layers, to adapt its weights, which can be accomplished using error backpropagation based on manually labelled body parts in a training image set. When subsequently presented with a novel image not part of the training set, the network then produces an output with its estimate of body part positions within that image. With suitable supervised or unsupervised postprocessing, estimated animal pose for each video frame can be used to classify behavioural states^47–49^, although this step can be far from straightforward and requires adaptation to the particular details of the experimental setting. With this approach, classified behaviours inside the home cage environment can be linked to neural activity in brain regions of interest. The advent of markerless pose estimation algorithms using deep learning thus opens up the possibility for novel investigations that had not been previously possible.

## Results

**Fig. 1:**
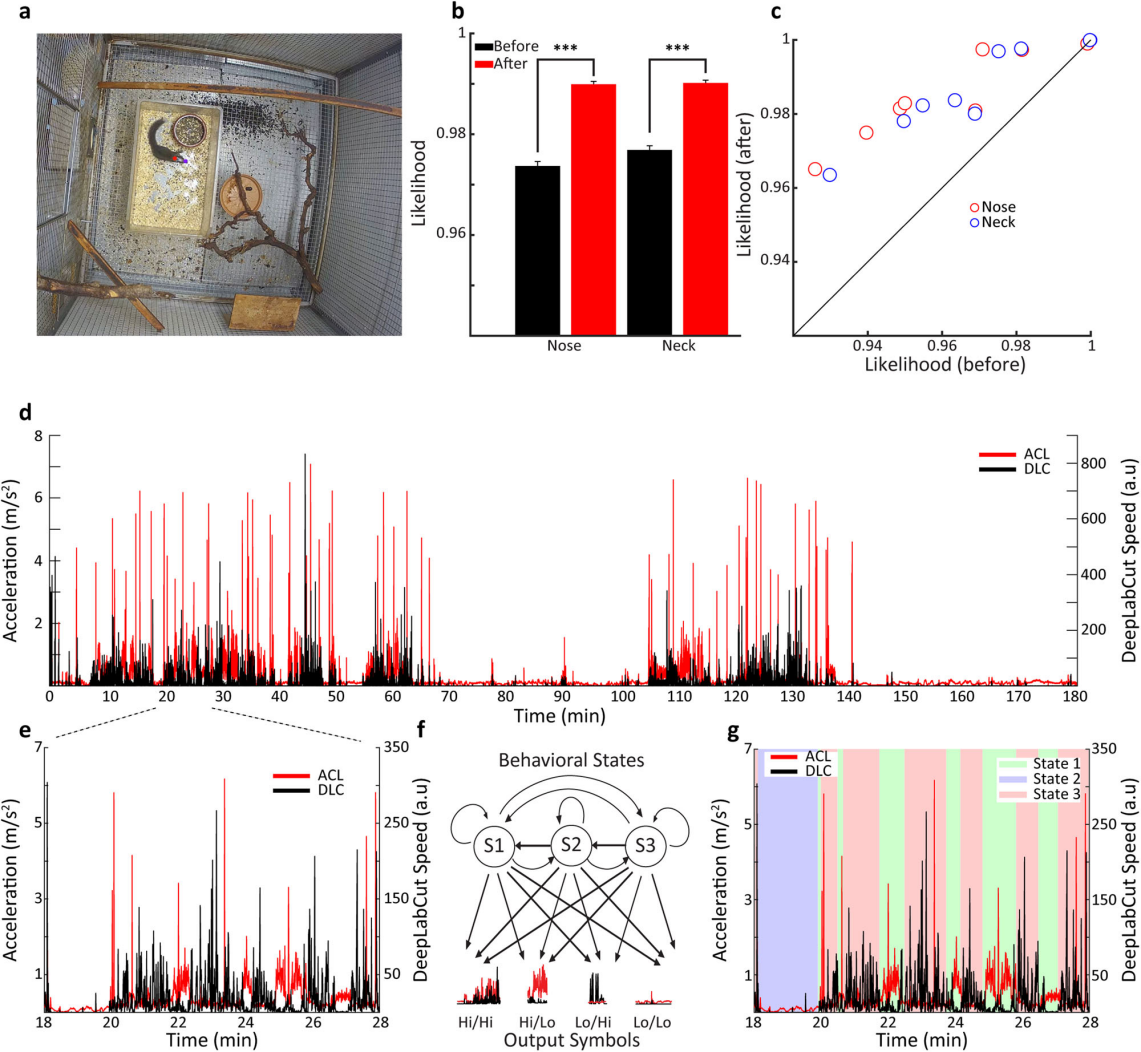
Segmentation of behavioural states. **a** Top view of the home cage environment showing a freely moving tree shrew equipped with the wireless recording device. The coloured points represent the body parts tracked by DeepLabCut. **b** DLC likelihood before and after relabelling of misclassified frames. Error bars represent SEM. **c** Scatter plot shows the likelihood before and after relabelling from 12 segments of a 4-h session. **d** A 3-h segment showing both accelerometer (ACL) and nose movement speed signals from DeepLabCut (DLC). **e** A 10-min segment taken from (**d**) with a detailed view of the complementary movement sensor signals ACL and DLC. **f** Hidden Markov Model with three states (S1, S2, S3) and four output symbols (Hi/Hi, Hi/Lo, Lo/Hi, Lo/Lo). **g** Three states assigned by Hidden Markov Model based on ACL/DLC input. Error bars represent standard error of the mean, SEM.

We first characterised tree shrew behaviour inside their home cage, a 3-m^3^ cage with multiple branches and other enrichment elements as well as access to food and water (Fig. 1a). Tree shrews were implanted with blunt tungsten microelectrodes (see ‘Methods’), targeting the DMN brain structures of interest, ventral pallidum (VP), anterior cingulate cortex (AC), mediodorsal thalamus (MD), and primary visual cortex (V1) as a non-DMN control. Local field potentials (LFPs) were registered with a wireless, battery-powered Neurologger device^50^. In addition to the LFPs, the Neurologger also registered 3-d accelerometer data, and since the Neurologger is connected to the head of the animal, the accelerometer registers sensitive information about high-speed head motion including vibrations, providing one useful indicator of behaviour exhibited by the tree shrew. To complement the accelerometer data, we obtained speed data from home cage video recordings using markerless pose estimation based on deep learning^46^. Based on continuous video recordings from a single camera mounted at the top of the cage, we manually labelled nose and neck in a limited number of video frames and then used Deeplabcut (DLC) to estimate the body part position for the remaining video frames (see ‘Methods’). The video was appropriately cropped and downsampled to 2 fps (ffmpeg, http://www.ffmpeg.org), allowing DLC network training to proceed on 20-min segments in a reasonable time of about 1–2 days for 500,000 iterations on available computing hardware including CUDA on a GEForce graphics card. The DLC analysis yielded the coordinates of the body parts and the corresponding likelihood estimates, where likelihood reflects the confidence of the network, between 0 and 1, that the predicted coordinates are accurate. For most of the frames, DLC accurately tracked the tree shrew, with likelihood values exceeding 0.99 and body part position corresponding to human observer estimations when viewing the video. For some frames, for example when the animal was partially occluded or in an unusual pose, DLC estimates appeared inaccurate, and the estimation needed refinement. We thus labelled an additional 50 frames where necessary and retrained the network for an additional 200,000 iterations. The mean likelihood of both body parts significantly increased after additional labelling (Fig. 1b, Wilcoxon signed-rank test, *P* < 0.001, nose: *P* = 3.7e^−10^, neck: *P* = 1.1e^−40^. *n* = 28,800 frames, from 12 video segments, 2400 frames each), and indeed position estimate likelihood generally increased notably except for cases where it was already close to 1.0 before additional labelling (Fig. 1c, Wilcoxon signed-rank test, *P* < 0.01, nose: *P* = 0.0093, neck: *P* = 0.0024. *n* = 12 video segments).

**Fig. 2:**
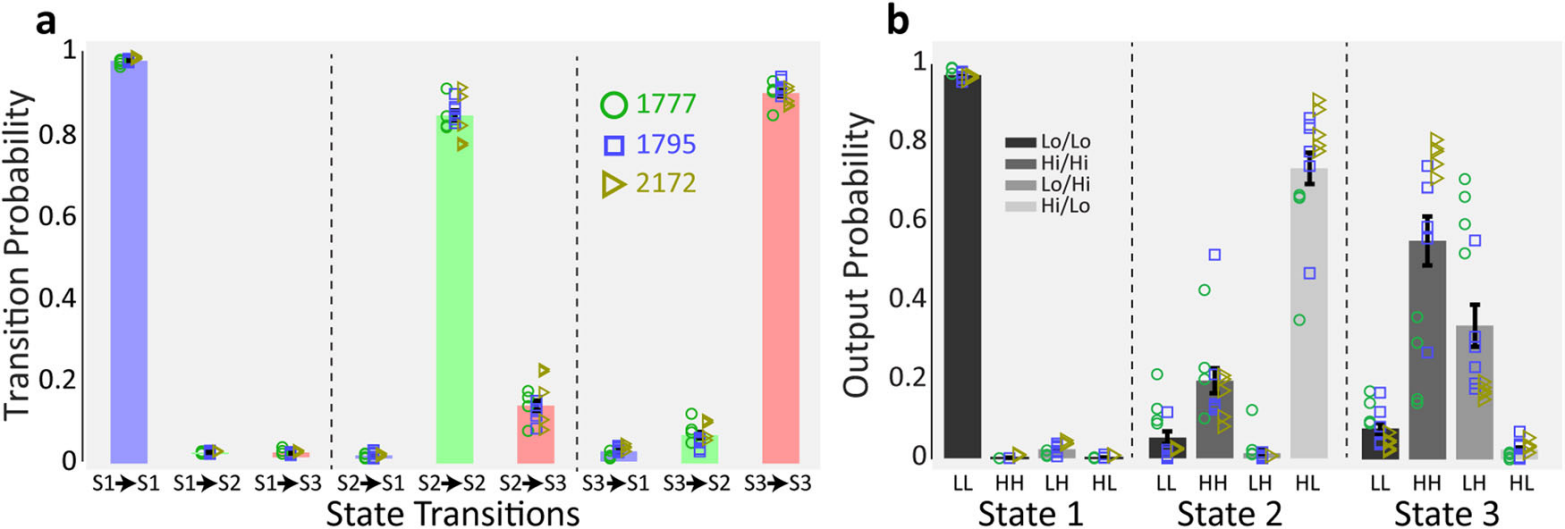
State transitions and output probability. **a** Group analysis of the transition probability for the Hidden Markov Model. **b** Group analysis of the output probability distribution of the Hidden Markov Model. Here, Lo/Hi refers to low ACL and high DLC activation. Error bars, SEM.

**Fig. 3:**
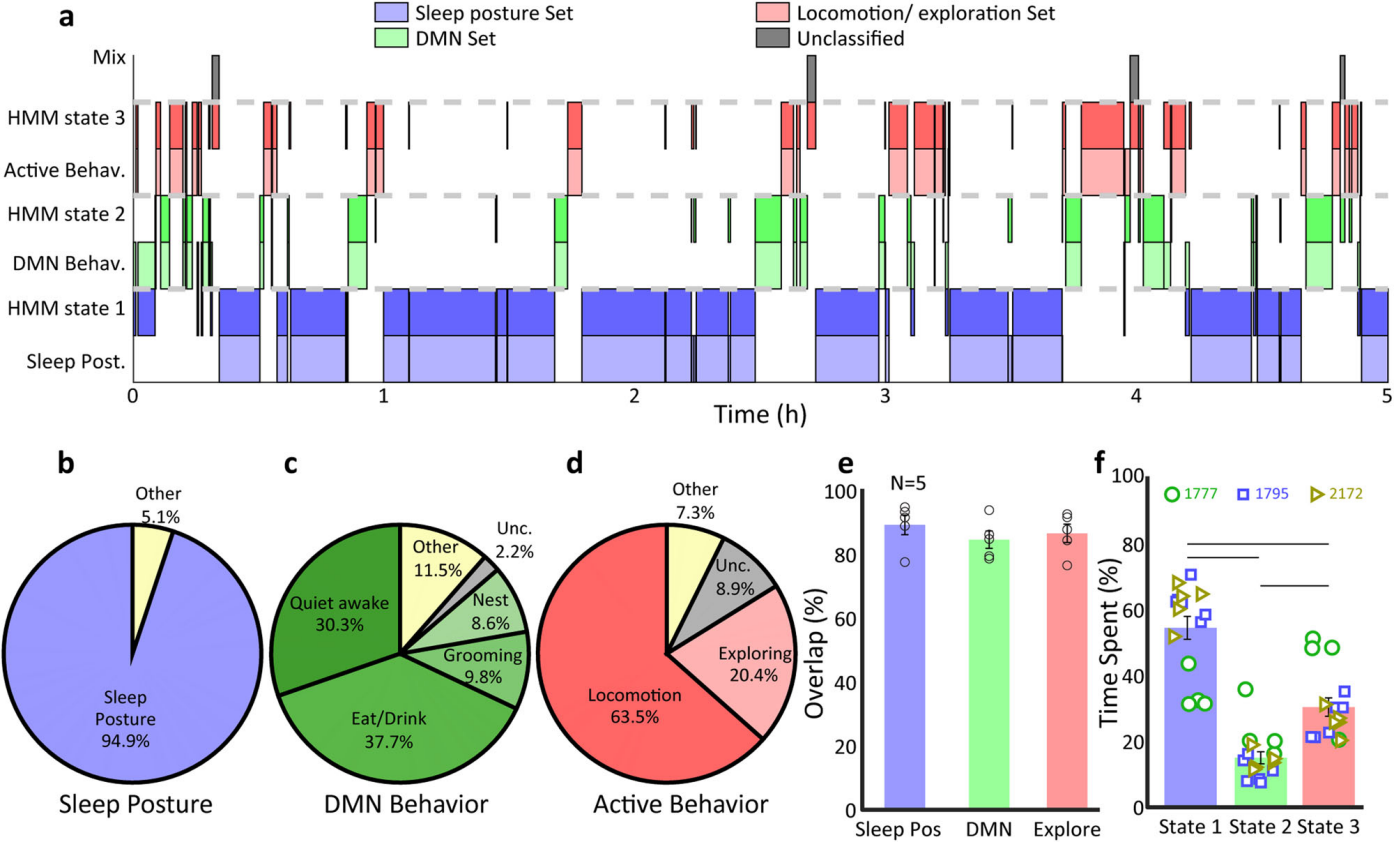
HMM classification performance. **a** Shows an example of the typically high correspondence between the states output by the HMM, and the behavioural sets derived from the manually labelled data. **b**–**d** Pie charts showing the composition of different behaviours observed in the 3 HMM states. **e** Mean overlap of the HMM output and manually scored behavioural sets. **f** Overall distribution of time spent in the different states revealed by the HMM for all animals and all sessions. Error bars reflect SEM. Lines represent *P* < 0.01.

From the position data, we estimated the speed of the animal as the difference between nose positions on subsequent frames, providing information related mostly to animal locomotion at a complementary temporal scale to the accelerometer data. Examining the accelerometer (ACL) and DeepLabCut (DLC) speed signals, we noted as expected generally similar activations over the course of the home cage recording period typically lasting about 6 h. Periods of low speed, corresponding to a stationary state alternate with periods of mobility (see Fig. 1d for a 3-h segment from an example tree shrew). Preliminary visual inspection revealed that low mobility periods encompassed mostly ACL-defined epochs where the tree shrew was typically curled up in a stereotypic sleeping posture, as well as quiet wakefulness where the tree shrew remained immobile in a more regular posture. High mobility periods contained the multiple active, ACL-defined behavioural states that tree shrews exhibited in their home cage, including locomotion, exploration, food and water consumption, grooming and quiet wakefulness. Examining the high mobility periods in more detail, we made an interesting observation, in that there were periods of conjoint high activation in both sensors as well as periods with high ACL but low DLC activation (see Fig. 1e). Preliminary visual inspection indicated that high/high ACL/DLC activation appeared to correspond mainly to locomotion or exploratory activities, whereas high/low ACL/DLC activation occurred during periods where the tree shrew was stationary but engaged in various activities including eating, drinking, as well as grooming and quiet wakefulness that we have considered previously as default mode-network (DMN) associated behaviours. This observation suggests that the ACL and DLC speed data provide complementary information about behavioural state, and in particular disjunctions between these signals appear to tag a specific set of behavioural states. We therefore decided to employ a hidden Markov model (HMM) for unsupervised classification of behavioural state based on ACL/DLC information (Fig. 1f). A key element of the HMM is the designation of the output symbols and number of states. Based on our preliminary observations, we designated four output symbols, corresponding to High/High, High/Low, Low/High and Low/Low combinations of ACL and DLC signal values. These output symbols cover the entire set of sensor readings, and it is ensured that each time point is associated with the emission of a single output symbol. The threshold between High and Low sensor values was determined based on the median value of the signal across the recording session. We selected a three state HMM, with the intention to capture the three groups of behavioural states, locomotion, DMN, and sleep. We used maximum likelihood estimation to find HMM state transition and output symbol emission probabilities and the Viterbi algorithm to compute the most probable state sequence given the estimated parameters. We found that the HMM was well able to capture the dynamics of the ACL/DLC sensor signals (Fig. 1g). The HMM parameters for this example session are summarised in Tables 1 and 2. As the probabilities were similar to those we observed in a population of 15 sessions in 3 tree shrews, we continue here to discuss directly the group data. For state transition probabilities (Fig. 2a), we found, as anticipated, that the probability for remaining in the same state was highest for all states (one-way ANOVA, *n* = 15 sessions, *P* < 0.05). The maximum probability 0.98 was seen for the 1 → 1 transition, yielding a mean dwell time for state 1 of 398 ± 31 s for the curled-up posture state. States 2 and 3 tended to have lower same state probabilities (one-way ANOVA, *n* = 15 sessions, *P* < 0.05), with mean dwell times of 47 ± 6 s and 72 ± 5 s, respectively. State 2 → 3 transitions were more probable than 3 → 2 transitions (one-way ANOVA, *n* = 15 sessions, *P* < 0.05), suggesting more frequent occurrence of the DMN-associated state followed by exploration than vice versa. In terms of output symbol probabilities (Fig. 2b), state 1 was almost exclusively associated with the Lo/Lo output symbol, as both movement sensors were inactive during the curled-up sleeping posture. State 2 was also associated with the Hi/Lo output symbol (0.74 ± 0.04) that tended also to be unique to this state, with some occurrence also of the Hi/Hi symbol. This symbol was indeed shared with state 3, where it co-occurred with symbol Lo/Hi that tended to be specific to this state. Notably, there was some animal-specific variation in output symbol probability particularly for state 3, such that in animal 1777 the state-specific symbol Lo/Hi occurred significantly more frequently than the other two animals. We consider that this probably reflects different behavioural patterns in this animal, which is consistent with the analysis described in Fig. 3 below. Overall, across animals, during active behaviours, each state tended to emit a mixture of output symbols suggesting some overlap on sensor activation patterns in these two states.

**Table 1:** Example HMM parameters 1

	To state 1	To state 2	To state 3
From state 1	0.98	0.01	0.01
From state 2	0.01	0.87	0.12
From state 3	0.04	0.06	0.90

Hidden Markov Model state transition probabilities.

**Table 2:** Example HMM parameters 2

	Lo/Lo	Hi/Hi	Lo/Hi	Hi/Lo
State 1	0.98	0	0.01	0.01
State 2	0.01	0.21	0	0.78
State 3	0.08	0.59	0.29	0.04

Output symbol emission probabilities. Lo/Hi refers to low ACL and high DLC activation.

To validate the results of the unsupervised behaviour classification using the HMM, we manually labelled each of the epochs identified by the HMM by visual inspection. Each HMM epoch was assigned one of seven labels: curled-up sleeping posture, quiet awake, eating/drinking, grooming, in or near nest box, locomotion and exploration. We parsed these labels into three behavioural sets. Behavioural set 1 represents sleep posture, while set 2 encompasses quiet wakefulness, eating/drinking, grooming and nest-box-associated behaviours. These set 2 behaviours are those with an internal focus that we have previously also linked to activation of the brain default mode network (DMN). Behavioural set 3 comprises locomotion and exploration, where attention is directed to external environment. By comparing the HMM states with these manually scored behavioural sets, taking one session as an example, we found that the HMM states indeed corresponded well to the sets of behaviours (Fig. 3a). Note however that the correspondence was not perfect, such that in a minority of cases the assignments diverged. Quantifying the overlap, we found that in 95% of HMM state 1 time periods tree shrews were in curled-up sleeping posture, 86% of HMM 2 segments were DMN-associated activities, and 84% of HMM 3 consisted of locomotion and exploration (Fig. 3b–d). Only a minority of between 5% and 16% of time segments did not directly correspond, such that there was a mismatch between HMM and manually scored behavioural sets. We confirmed these findings for a single example session across a set of 5 videos from 3 tree shrews that were processed using DLC/HMM analysis as well as manually classified by visual inspection. In particular, HMM and behavioural sets corresponded to each other for over 80% of respective time periods (Fig. 3e, one-way ANOVA test, *P* = 0.5431, *n* = 5 datasets). Taken together, this analysis shows that HMM states largely capture behaviours of the three sets, allowing us to proceed with the HMM state assignment for a larger number of datasets without arduous, manual annotation. In the 15 sessions of video classified by HMM, we found out that on average, 54 ± 3% of time the tree shrews are in the HMM state corresponding to sleeping posture, 15 ± 2% in the HMM state corresponding DMN activation, and 30 ± 3% in the HMM state corresponding to locomotion/exploration (Fig. 3f, one-way ANOVA test, *P* = 1.9e^−13^, *n* = 15 sessions). The behavioural patterns for the tree shrews were similar to each other; but note that animal 1777 tended to spend less time in sleeping posture and more time in exploration behaviours.

**Fig. 4:**
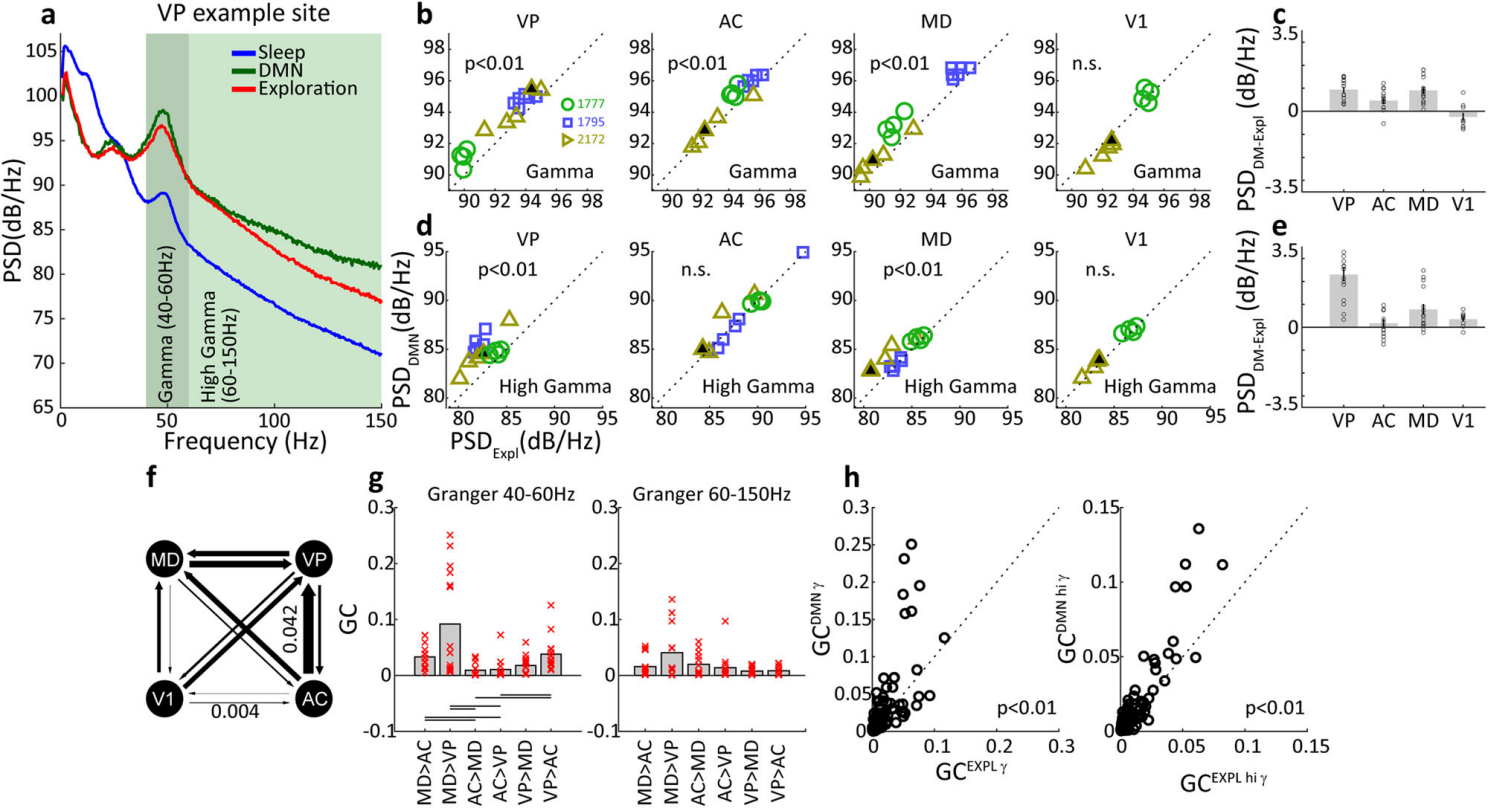
LFP Gamma power and its relation to behaviour across brain areas. **a** Example PSD of LFPs recorded from a single animal in the VP during the three HMM-derived states. **b** Scatter plots showing the spectral power at gamma frequencies (40–60 Hz) during DMN-related behavioural activity vs exploratory behaviours in the four brain areas. Axes are the same as in (**d**). Symbol colours denote different animal subjects, see the legend. **c** Average magnitude of the difference in gamma power between DMN and exploration behaviours in the four brain areas. Error bars depict SEM. **d**, **e** same as (**b**, **c**) but for the high-gamma band (60–150 Hz). **f** Average directional interaction strength (Granger causality) calculated for the gamma band (40–60 Hz) between VP, AC, MD, V1 during DMN-related states. Line thickness proportional to the magnitude of the Granger Causality. **g** Directional interactions among DMN-related sites (VP, AC, MD) during DMN-related states for gamma, left, and high-gamma band, right (lines represent *P* < 0.01). **h** Scatter plots comparing overall Granger causality values within VP, AC and MD between DMN and exploratory behavioural states. Error bars depict SEM.

**Fig. 5:**
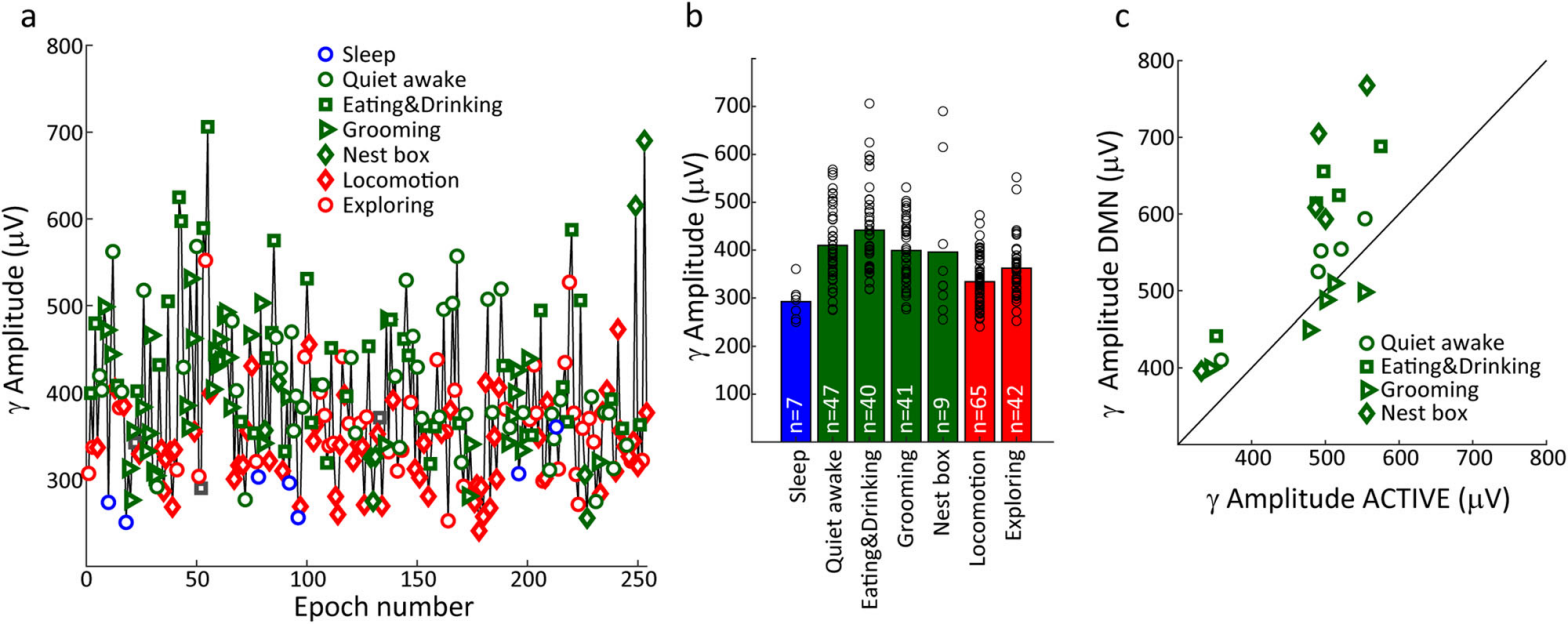
Gamma power during individual behaviours. **a** Example of average gamma power (40–60 Hz) for behavioural epochs in chronological order throughout a single session. **b** Average gamma amplitude of all epochs for the different behaviours in the session in (**a**). **c** Scatter plots showing gamma amplitude during each of the DMN-related behaviours vs gamma power during active states (Locomotion and Exploration) in all five manual labelled datasets.

We proceeded to analyse the local field potential (LFP) data registered at the four brain sites: ventral pallidum (VP) of the basal forebrain, mediodorsal (MD) nucleus of the thalamus, anterior cingulate (AC) cortex and primary visual cortex (V1), which is not part of the DMN and serves as a control brain region in the present study. An example LFP power spectrum recorded in VP is shown in Fig. 4a for the three HMM-defined behavioural states. We note prominent modulations in the gamma range that were associated with behavioural state, such that spectral power in both the gamma (40–60 Hz) and high gamma (60–150 Hz) bands was largest during DMN behaviours, attenuated during exploration and strongly attenuated during sleep. Comparing gamma activity during DMN and explorative behaviours, we found that the attenuation was indeed significant in VP as well as the other DMN brain areas MD and AC (*t* tests, *P* < 0.01, VP: *P* = 1.5e^−6^, AC: *P* = 0.0011, MD: *P* = 1.6e^−5^ *n* = 15 sessions) but not in V1 (Fig. 4b, c, *t* tests, *P* = 0.1967, *n* = 9 sessions); note that V1 data was available for only two animals. Generally similar results were found in the high-gamma band (Fig. 4d, e), with significant attenuation during exploration relative to DMN behaviours observed in VP and MD, but not in V1 or AC. Taken together, VP, MD and AC exhibit elevated gamma oscillations during DMN-associated behaviours compared to active exploration, with high-gamma modulations occurring specifically in the subcortical areas. This finding extends previous observations of elevated gamma activity in DMN nodes VP and AC of the rat to the tree shrew^20^, and strongly implicates the MD thalamus as a participant node of the DMN. To examine directional interactions between the recording sites, we used Granger causality modelling, which allows us to compute to what degree past values of one timeseries can predict future values of another timeseries. We first examined all 12 possible gamma-band directional interactions during the DMN behavioural state among the four recording sites (*n* = 9 sessions), which revealed that 6 interactions among DMN-related sites (VP, MD, AC) were overall larger than 6 interactions between V1 and the DMN sites (see Fig. 4f, Wilcoxon test, *P* = 0.0039). This finding validates specific enhancement of functional coupling among DMN brain areas. Focusing then on interactions within the DMN-related sites, we used a Kruskal–Wallis non-parametric ANOVA to examine if there were significant differences between the interactions. We found that three gamma-band interactions were particularly elevated, namely MD → AC, MD → VP and VP → AC (see Fig. 4g, left. Kruskal–Wallis test, *P* = 1.7e^−6^, *n* = 15 sessions), whereas for the high-gamma band none of the Granger directional influences differed significantly (see Fig. 4g, right. Kruskal–Wallis test, *P* = 0.4722, *n* = 15 sessions). For Granger causality, we also wanted to examine if these differed within DMN sites between behavioural states. Here, we grouped all six values together and found overall that in both gamma and high-gamma band, Granger causality values were significantly enhanced during DMN behavioural states compared to the locomotion/exploration behavioural state (see Fig. 4h, *t* tests, *P* < 0.01, gamma band: *P* = 0.0034, high-gamma band: *P* = 0.002. *n* = 90, 6 values from 15 sessions). This is consistent with the idea that the DMN brain network nodes VP, MD and AC are engaged during DMN behavioural states. The above analysis focused on oscillatory brain activity in the automatically labelled behavioural states identified using markerless pose estimation combined with HMM classification. For the five datasets with manual scoring, we repeated the analysis to examine brain activity among the individual behaviours contributing to each HMM-identified state. Results for VP in an example session are shown in Fig. 5a, illustrating gamma activation during the chronological progression through the behavioural states. Note that gamma amplitude tended to be low during sleep posture and appeared elevated during DMN-related behaviours; a tendency that was confirmed by examining mean gamma amplitude across all occurrences of the respective behaviour (Fig. 5b). Analysing all five available datasets with manual scoring (Fig. 5c), we found that gamma activation was significantly elevated for quiet wakefulness, eating/drinking, and nest-box proximity (*n* = 5 for each genre of activity, paired *t* test, *P* < 0.05. Quiet wakefulness: *P* = 0.001, eating/drinking: *P* = 1.5e^−4^, nest box: 0.0127) but not for periods of grooming (paired *t* test, *P* = 0.5770).

**Fig. 6:**
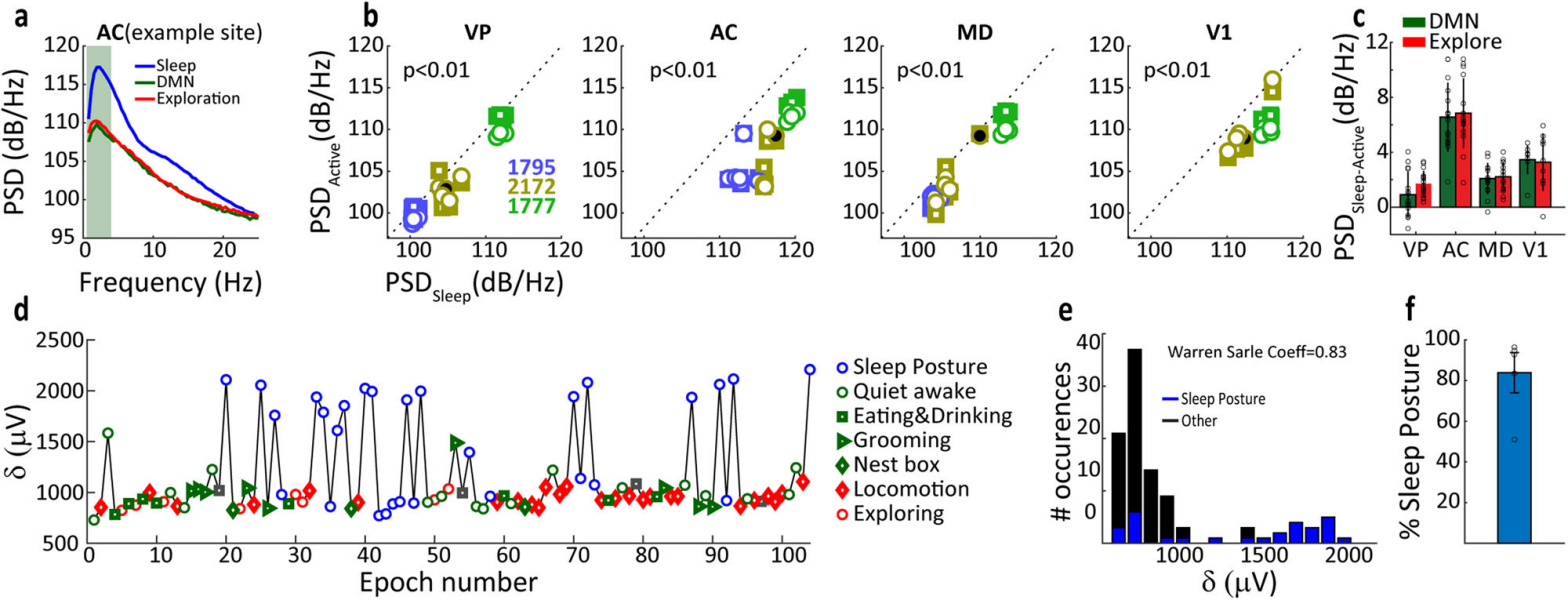
Delta band activity and behaviour. **a** Example of LFP power spectral density (PSD) in AC for three behavioural states. **b** Scatter plots showing delta band (0.5–4 Hz) PSD during sleep posture vs active states (including DMN and Exploratory states) in the four brain areas. Symbol colours denote different animals, see the legend. **c** Average magnitude of the difference between delta band PSD during sleep posture and two active states (DMN and Exploration). **d** Average delta amplitude of each epoch in chronological order throughout one session. **e** Warren–Sarle test of bimodality showing the delta amplitude distribution of sleep posture epochs was bimodal for the example session. (Warren–Sarle coefficient = 0.83). **f** Across the datasets, the percentage of sleeping posture in epochs with high delta activity occurred. Error bars reflect SD.

**Fig. 7:**
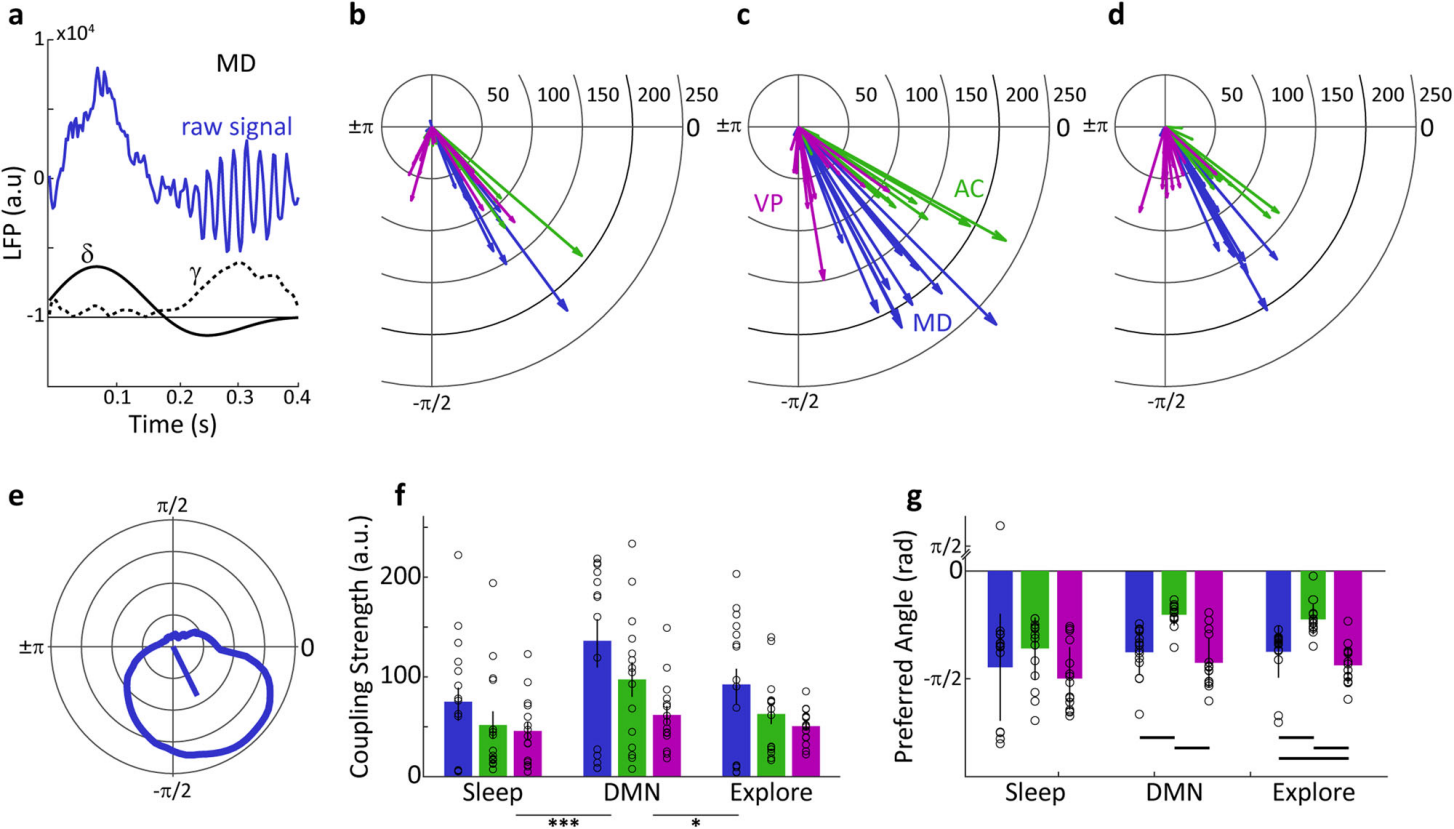
Delta–gamma coupling across brain areas. **a** A raw segment of the LFP taken from MD thalamus with prominent delta and gamma activity is shown at top, at the bottom the amplitude of delta and gamma power for the same segment. **b** The delta–gamma cross-frequency coupling in polar format. The cfc vector is computed to the centroid of the angular cfc distribution. **c**–**e** The cfc vector distribution for sleeping posture (**c**), DMN behaviours (**d**) and locomotion/exploration (**e**) in MD, AC and VP. **f** Cfc coupling strength in MD, AC and VP (two-way repeated-measures ANOVA, ***P < 0.001, *P < 0.05, error bars indicate SD). **g** Cfc preferred angle in MD, AC and VP (error bars indicate SD, lines represent *P* < 0.01).

In addition to gamma modulations, robust variations in delta band activity (0.5–4 Hz) were evident in our recordings. An example of delta power during the three HMM states is shown in Fig. 6a, illustrating enhanced delta power during sleeping posture, which is expected since prominent delta waves occur during slow-wave sleep^44^. The effects were largest in the AC cortex, but also evident in V1 and the subcortical regions (Fig. 6b, c). For an example session, we examined delta amplitude during manually labelled states (Fig. 6d) and found that only a subset of the sleep posture epoch actually corresponded to slow-wave sleep, with delta amplitude reaching around 2 mV. During the remaining sleep posture epochs, delta amplitude was similar to other behaviours, suggesting that the tree shrew was probably engaged in rapid eye movement sleep. A Warren–Sarle test of bimodality showed that delta amplitude distribution was indeed bimodal for this example dataset (Fig. 6e), and that across datasets the tree shrew was in sleeping posture in over 80% of epochs where high delta activity occurred (Fig. 6f, threshold of high delta activity defined by Min + (Max-Min)/2). Delta and gamma activity often occur in a coordinated manner in the cortex, and subcortical regions, and the relationship between gamma power and phase of the delta cycle can provide useful information about local circuit activations. An example of delta–gamma coherence recorded in the MD thalamus is shown in Fig. 7a for a representative delta cycle, illustrating that gamma activity tended to occur in the negativity/trough of the delta cycle and also during the rising phase. Figure 7b shows the delta–gamma cross-frequency coupling (cfc) in polar format, with the cfc vector computed to the centroid of the angular cfc distribution. For this delta cycle, the preferred angle occurred at a delta phase of about -π/4. In Fig. 7c–e, cfc vectors are shown across the 15 datasets from 3 tree shrews for sleeping posture, DMN behaviours and locomotion/exploration respectively. Cfc coupling strength was largest in MD thalamus, intermediate in AC and smallest in VP (two-way repeated-measures ANOVA, ***P < 0.001, *P < 0.05, main effects of brain area and behavioural state and significant interaction, see Fig. 7f). At the same time, cfc coupling strength was greatest during DMN behaviours, intermediate during locomotion or exploration and smallest during sleeping posture. The low cfc coupling strength during sleeping posture is consistent with the overall low prevalence of gamma activity in this behavioural state. The analysis of cfc preferred angle^51^ revealed interesting differences between the three brain regions, with gamma activity occurring earliest in the delta cycle in VP, followed by MD and finally by AC during exploration as well as DMN behaviours (Circular Median test, *P* < 0.01, see Fig. 7g). No significant difference in cfc preferred angle were seen during sleeping posture. These findings suggest that the local relationship between delta cycle and gamma activation varies between brain regions, with gamma activations tending to occur earliest in VP, and later in MD and AC with respect to the local phase of the delta cycle.

## Discussion

Our findings show that tree shrew basal forebrain nucleus VP exhibits robust oscillations in the gamma range (40–60 Hz), that are enhanced during behavioural states associated with the DMN compared to non-DMN states such as locomotion and exploratory behaviours. This extends previous findings in the rat^19,52,53^ that have demonstrated enhanced gamma oscillations in VP and adjacent ventral striatum during quiet immobility compared to locomotive behaviour, underscoring that in both mammalian species, the VP can be considered as a subcortical DMN node. This evidence corroborates results from human fMRI studies that have also implicated the basal forebrain as an important subcortical component of the DMN^24^. Causal evidence from optogenetic activation of the basal forebrain using optogenetics further supports its important role in DMN regulation^27,54^. We also observed elevated VP gamma activity during food and water consumption, compatible with previous data suggesting a role for VP in feeding behaviour. The medial VP receives a direct GABAergic projection from the shell region of the nucleus accumbens (AcbSh), and sends a direct GABAergic projection to the lateral hypothalamus. Both deactivation of AcbSH, and pharmacological blockade of GABA_A_ receptors in VP lead to a marked increase in feeding activity, with the AcbSh-mediated effects abolished by lesions of the medial VP^55,56^. Further, gamma oscillations in the ventral striatum, including accumbens, have been directly linked to food consumption, with these oscillations carrying multiple types of food-related information^57^. There is also evidence for the regulation of drinking by forebrain nuclei. Water intake and homoeostasis are controlled by lamina terminalis structures including the median preoptic nucleus^58^, which is in close proximity to our VP recording site and contains a coupled network of glutamatergic and GABAergic neurons that might potentially contribute to the generation of gamma oscillations^59^, although this remains to be demonstrated. Finally, previous reports in humans have shown diminished DMN activity and interconnectivity during both bulimia and anorexia-type eating disorders^60,61^, suggesting the possible connection between the DMN activity and the regulation of food intake. As for self-grooming, we observe here a discrepancy between rat and tree shrew. Whereas grooming is associated with large amplitude gamma oscillations in rat VP, no such effect was seen in the tree shrew in the present study. Since grooming can help regulate stress^62^, this difference in DMN regulatory mechanisms might contribute to the particular sensitivity to stress that tree shrews tend to exhibit^63^. We speculate that grooming in the tree shrew may fail to activate the DMN as it does in other species, and that reduced DMN activation and interareal coherence might have a negative impact on stress regulation in this species^64^. This hypothesis is supported by human neuroimaging showing that following a highly stressful experience, a major earthquake, there is a decrease in functional coupling between the VP and cortical DMN nodes including posterior cingulate and precuneus cortical areas^65^ It is interesting to note that rodents that receive less maternal grooming, show heightened stress responses^66^, and that unlike rodents, maternal care, including grooming, is largely absent in the tree shrew. Tupaia mothers practice absentee maternal care, spending very little time with their pups and only returning to the nest to nurse their young for roughly 5 min every 48 h. During these brief nest visits mother/pup grooming is not observed, presumably due to time constraints^67^. Together with emerging evidence, our study thus supports the important role of the basal forebrain VP and related structures in activating the DMN and triggering associated behavioural states. Across species, these DMN-associated brain states include inward-focused quiet wakefulness and occupancy of a highly familiar nest-box environment, as well as consumption of food and water when it is freely available as in our study, but perhaps not in an exploratory or active foraging context.

Our experimental findings confirm the involvement of MD thalamic nucleus in DMN regulation in the tree shrew, as has been shown in human neuroimaging and dynamic causal modelling^24,25^. Our observations of enhanced activations in gamma and high-gamma bands during DMN-associated behaviours in MD, as well as enhanced Granger causal coupling among DMN brain areas during these behaviours both support this conclusion. Indeed, significant Granger coupling from the mediodorsal thalamus to the frontal cortex has previously been observed in humans with intracranial implanted electrodes^68^. Harrison and colleagues concluded that the basal forebrain impact on the DMN is likely direct, rather than mediated via the MD, but that MD serves to stabilise and coordinate DMN cortical activations. This is consistent with two aspects of our gamma-band Granger causality modelling: first, we find a significant bi-directional modulation between VP and anterior cingulate (AC) cortex underscoring the importance of the direct influence of basal forebrain VP nucleus on DMN cortex. Second, an elevated modulation from MD to VP compared to the reverse direction, favouring the view that MD is more crucial in coordinating and stabilising activations rather than broadcasting VP activity to DMN cortex. AC was chosen as a cortical DMN region of interest as it has been shown to be a part of the DMN in rats, monkeys and humans^9,69,70^, it has a strong reciprocal connection with the MD of tree shrews^32,71^, and is tightly correlated with VP activity during DMN states in the rat^19,27^. While a direct inhibitory projection from VP to MD is well documented^72,73^, the reverse projection appears to be polysynaptic; as we did not find evidence for a direct projection in the literature. Nevertheless, our findings and those of Harrison and colleagues suggest a significant coordinated functional influence of MD and VP on the DMN. The importance of thalamic influence on the DMN is supported by findings in patients suffering from mild cognitive impairment, where white matter integrity from thalamus to DMN cortical areas is compromised, along with reductions in Granger causal influence from thalamus to these DMN regions^74^, This is consistent with emerging evidence suggesting that thalamic circuits play a more important role than previously acknowledged^75^, in structuring and coordinating cortical activations. Furthermore, our results are compatible with the notion that medial thalamic nuclei exert control over distributed cortical networks and mediate alternating activations of the DMN and task-positive networks^22,76^. The presence of gamma oscillations in MD in a DMN context has not been documented to our knowledge. However, there is some evidence compatible with this idea. For example, walking on a treadmill, which we consider as a potential DMN behaviour, as it is highly repetitive and unlike exploration or regular locomotion does not require attention to the environment, elicits gamma oscillations in MD^77^. More generally, current thinking about the MD thalamus revolves around the notion of cortical network regulation^78^, compatible with a role in the DMN.

In addition to the regular gamma-band effects (40–60 Hz), we also document state-dependent changes in the high-gamma (60–150 Hz) band that are largely consistent with the regular frequency gamma band but that were distributed in nature, and lacked a specific peak frequency. We did find that high gamma tended to be more pronounced in subcortical brain regions MD and VP rather than in cortical brain regions. Not much has been reported about high gamma in the subcortical regions we investigated here, but high gamma has been observed in visual cortex where it is known to be more closely related to spiking activity than regular gamma activity^79,80^. A closer connection of high gamma to local spiking activity is indeed compatible with our own observation that no long-range Granger causality modulations among DMN nodes reached statistical significance. Future studies incorporating the recording of spiking activity in cortical and subcortical DMN structures and their relation to the different gamma-band activations are needed to confirm this idea and to reveal in more detail the nature of information flow within the DMN. We also analysed delta oscillatory activity (0.5–4 Hz), and these were, as expected, most pronounced in AC cortex. Since our behavioural scoring was related to sleeping posture in the present study, we had no way of distinguishing different sleep states during the epochs that tree shrews spent in this posture. Examining AC delta in more detail, confirmed that in a subset of these epochs tree shrew were engaged in slow-wave sleep, whereas other epochs might correspond to REM sleep or another wakeful or drowsy states. We have reported details of overnight sleep brain activity dynamics in tree shrews previously^44^, and focus here on the various activities that occur during wakefulness. Nevertheless, our findings show that our animals in the housing facility do spend a significant fraction of the day engaged in slow-wave sleep, in contrast to wild tree shrews that are almost continuously engaged in foraging behaviours when they leave their nest during daytime^81^. Finally, we document a striking relationship between the amplitude of gamma oscillations and the phase of local delta oscillations at the different DMN sites. While during sleeping posture, cross-frequency coupling strength and preferred angle were similar across brain sites, we found significant differences during wakeful behaviours in terms of coupling strength and phase preference, such that VP gamma occurred early in the delta cycle, followed by MD and AC late in the cycle. Cross-frequency coupling has been previously observed in cortical and subcortical brain circuits^82^, and it is thought to organise and structure information flow and inter-site communication between distant brain structures.

Our study is part of a rapidly advancing literature studying brain activities in freely moving animals in conjunction with pose estimation, which promises to deliver unprecedented insights into neural mechanisms underlying physiological and cognitive functions^83^. The novel algorithms based on residual neural networks are useful and can be adapted to a wide range of tasks ranging from high-resolution tracking of limb kinematics to tracking of animal position in cluttered environments^45,84^. Our application falls into the latter category and is distinct from work in rodents in that tree shrews navigate, often at high speed, in a large and complex three-dimensional home cage environment containing multiple enrichment elements, where the animal is frequently partially occluded. On the other hand, rodent work typically involves monitoring in much smaller enclosures that often do not contain substantial three-dimensional structure^47^. Our setup involves a single wide-angle camera mounted in the centre of the cage ceiling, such that we obtain only video material of a limited resolution, particularly when the animal is on the cage floor. For this reason, we employed pose estimation with relatively low spatial and temporal resolution and combined the video-based estimates with accelerometer data available from the Neurologger device^85,86^ that was also for recording neural activations. Our study thus combined video-based and accelerometer-based information regarding tree shrew behavioural state, which proved very useful as the two types of sensor data provide complementary information. While a variety of tools are becoming available for classifying behavioural state based on pose^48,49^, we used a simple hidden Markov model (HMM) to combine the two types of available sensor data in our application. This approach was successful in terms of reliable performance on our datasets in assigning behaviours into the three groups of sleeping posture, DMN-associated, and exploration/locomotion. For classification among behaviours within the groups, we did resort to manual scoring, but this process was much facilitated by the segmentation done in the pose estimation algorithm. Overall, our experience suggests that the new algorithms do permit the discovery of functional relationships between brain activations and behaviour, but good results are obtained only with substantial effort and expertise. We feel that cooperation across laboratories will be key in the future to develop robust methods of behavioural classification for particular species and experimental settings.

## Methods

The local ethical committee on animal experimentation (canton of Fribourg), approved all experimental procedures.

### Animals

Three adult tree shrews, *T. belangeri*, of either sex were housed under a 13/11 LD cycle in a 3-m^3^ cage with branches, some enrichment elements, and ad libitum access of food and water. The cage was connected to a nest box with a tube.

### Surgical procedures

Animals first received i.m. injections of Alfaxan (40 mg/kg) to induce anaesthesia and Atropine (0.08 mg/kg) to prevent secretions. Animals were then intubated using a modified otoscope (Bebird, Alhambra, CA), ventilated at 100 bpm (Small Animal Ventilator, Harvard Apparatus, Cambridge, MA), and placed in a stereotactic frame (David Kopf Instruments Tujunga, CA). Anaesthesia was maintained with isoflurane (1–3%) in pure oxygen, and end tidal CO_2_ was monitored (Physiosuite, Kent Scientific Torrington CT) and maintained at ~4%. Lidocaine (0.5 ml 1%) was injected near the incision site, a midline incision was made and the skull was exposed. Three 1.5 mm stainless-steel bone screws (WPI Hertfordshire, UK) were implanted with two located above the cerebellum as a reference and ground. Burr holes were drilled, and epoxy coated tungsten electrodes (FHC Bowdoin ME) with a tip resistance ~150 kΩ were lowered to the recording sites: Ventral pallidum (AP 7.6 mm, ML 3.0 mm, DV −7.8 mm), anterior cingulate cortex (AP 11.1 mm, ML 0.8 mm, DV −1.5 mm) mediodorsal thalamus (AP 4.9 mm, ML 1.0 mm, DV −5.4 mm) and primary visual cortex (AP 2.0 mm, ML 1.4 mm, DV −1.0 mm). All coordinates are from the interaural line. Electrodes were fixed to the skull with super glue (LOCTITE, Westlake OH) and Paladur dental cement (Kulzur Inc. Hanau Germany). Electrodes were wired to a socket connector, and the connector was attached to the skull with dental acrylic. The incision was closed about the connector with sutures, and the animal was allowed to recover for at least one week prior to testing.

### Data acquisition

LFP and accelerometer data were collected using a wireless battery-powered data logger (Neurologger 2A, Zürich Switzerland). Additionally an infrared receiver on the neurologger was used for aligning the LFP and accelerometer data with video recordings. All channels of neural signals and accelerometer data were digitised at 400 Hz and no further filtering was performed on the LFP data.

### Home cage recording

Video recordings of the animals in their home cage were made using a wide field, 103° × 58°, CMOS camera (DS-2CD2143G0-IS, HIKVISION, Hangzhou China) mounted on top of the cage. After connecting the Neurologger, tree shrews were initially kept in their nest box for 10 min in order to acclimate to the Neurologger device. Home cage recordings typically lasted for 5–6 h, between 9:00 and 18:00 during the animals’ perspective daytime.

### DeepLabCut tracking

DeepLabCut (DLC)^46,84^ was used track the animal’s location in the home cage. First, videos were pre-processed by cropping appropriately and downsampling to 2 fps, and then cut into 20-min segments. The model was trained for 500,000 iterations after manually labelling 50 frames from each video segment. For each frame, we labelled the nose and neck. The output consisted of body part coordinates in *x*, *y* coordinates and the corresponding likelihood estimate, using the same model to analyse all the video segments from the same animal. In most circumstances, DLC can accurately track the tree shrews in most of the frames. For those videos with obvious missing frames, an additional 50 frames were hand-labelled, and the network was retrained with additional 200,000 iterations.

### Preprocessing and spectral analysis

We partitioned the LFP data into 0.5-s epochs for further analysis. Power spectra were calculated by fast Fourier transforms (FFT). We calculated the band power by calculating the mean value of the power spectrum between 40 and 60 Hz (gamma band) and 60 and 150 Hz (high-gamma band).

### Hidden Markov model

We designed an HMM with three states to capture the three groups of behavioural states detailed in the results, and four output symbols corresponding to High/High, High/Low, Low/High and Low/Low combinations of ACL and DLC signal values based on preliminary observations of our data. The thresholds between High and Low sensor values were determined based on the median value of the signal across the recording session. We used maximum likelihood estimation to find HMM state transition and output symbol emission probabilities and the Viterbi algorithm to compute the most probable state sequence given the estimated parameters.

### Granger causality

To test the information transfer between VP, AC, MD and V1 brain regions, we used LFPs and a multivariate linear vector autoregressive (VAR) model from Matlab Multivariate Granger Causality (MVGC) toolbox^87^ for granger causality analyses. The maximum model order for model order estimation was 20 ms, and Akaike information criteria (AIC) was used. The model parameter for the VAR model estimation was the locally weighted linear regression (LWR). We used F-testing with a false discovery rate (*Q*  <  0.05) for the pairwise conditional Granger causality estimation. Kruskal–Wallis test was applied to determine the significance between the information transfer directions. *T* test was applied to compare the significance of Granger causality between the DMN state and the exploration state.

### Cross-frequency coupling

Cross-frequency coupling is estimated by accumulating the amplitude of gamma for each phase of the delta cycle, i.e. phase-amplitude coupling. First, the LFP is filtered into delta (0.5–4 Hz) and gamma (40–60 Hz) bands using a Butterworth band pass filter. The delta-filtered data is then transformed into a continuous series of phase angles using the Hilbert transform, while the gamma-filtered data is transformed into a continuous series of gamma amplitude values, also using the Hilbert transform. Finally for each point in the vector of delta phases, we accumulate the corresponding gamma power at that phase, resulting in the magnitude of gamma power for each phase of the delta cycle.

### Statistical analyses reproducibility

Experiment details are provided in the text and “Methods”. All the statistical analyses were performed in Matlab. In comparison of likelihood in Fig. 1, we applied the non-parametric Wilcoxon signed-rank test as the data did not follow a normal distribution. For the same reason, to compare granger causality values within VP, AC and MD, we used Kruskal–Wallis test (Fig. 4g). For normally distributed data with equal variance, *t* test or ANOVA tests were applied according to group number. In Fig. 6e, we applied the Warren–Sarle test in order to assess the bimodality of the delta band amplitude distribution for sleep posture epochs. In Fig. 7g, we used the Circular Median test (Matlab CircStat Toolbox) for a non-parametric multi-sample test of equal medians for circular data.

### Reporting summary

Further information on research design is available in the Nature Portfolio Reporting Summary linked to this article.

### Supplementary information

Reporting Summary

## References

[1] Shulman, G. L. et al. Common blood flow changes across visual tasks: II. Decreases in cerebral cortex. *J. Cogn. Neurosci.* **9**, 648–663 (1997). 23965122 10.1162/jocn.1997.9.5.648

[2] Menon, V. 20 years of the default mode network: a review and synthesis. *Neuron* **111**, 2469–2487 (2023). 37167968 10.1016/j.neuron.2023.04.023 PMC10524518

[3] Raichle, M. E. The brain’s default mode network Raichle ME. The brain’s default mode network. *Annu Rev. Neurosci.* **38**, 433–447 (2015). 25938726 10.1146/annurev-neuro-071013-014030

[4] Smallwood, J. et al. The default mode network in cognition: a topographical perspective. *Nat. Rev. Neurosci.*10.1038/s41583-021-00474-4 (2021). 10.1038/s41583-021-00474-4 34226715

[5] Barnett, A. J. et al. Intrinsic connectivity reveals functionally distinct cortico-hippocampal networks in the human brain. *PLoS Biol.* **19**, e3001275 (2021). 34077415 10.1371/journal.pbio.3001275 PMC8202937

[6] Li, J., Chen, J., Kong, W., Li, X. & Hu, B. Abnormal core functional connectivity on the pathology of MDD and antidepressant treatment: a systematic review. *J. Affect. Disord.*10.1016/j.jad.2021.09.074 (2022). 10.1016/j.jad.2021.09.074 34688026

[7] Jiang, S., Li, H., Liu, L., Yao, D. & Luo, C. Voxel-wise functional connectivity of the default mode network in epilepsies: a systematic review and meta-analysis. *Curr. Neuropharmacol.* **20**, 254 (2022). 10.2174/1570159X19666210325130624 PMC9199542 33823767

[8] Harikumar, A., Evans, D. W., Dougherty, C. C., Carpenter, K. L. H. & Michael, A. M. A review of the default mode network in autism spectrum disorders and attention deficit hyperactivity disorder. *Brain Connect.*10.1089/brain.2020.0865 (2021). 10.1089/brain.2020.0865 PMC8112713 33403915

[9] Lu, H. et al. Rat brains also have a default mode network. *Proc. Natl. Acad. Sci. USA* **109**, 3979–3984 (2012). 22355129 10.1073/pnas.1200506109 PMC3309754

[10] Hsu, L. M. et al. Constituents and functional implications of the rat default mode network. *Proc. Natl. Acad. Sci. USA* **113**, E4541–E4547 (2016). 27439860 10.1073/pnas.1601485113 PMC4978262

[11] Mantini, D. et al. Default mode of brain function in monkeys. *J. Neurosci.* **31**, 12954–12962 (2011). 21900574 10.1523/JNEUROSCI.2318-11.2011 PMC3686636

[12] Barks, S. K., Parr, L. A. & Rilling, J. K. The default mode network in chimpanzees (pan troglodytes) is similar to that of humans. *Cereb. Cortex* **25**, 538–544 (2015). 24046078 10.1093/cercor/bht253 PMC4303805

[13] Garin, C. M. et al. An evolutionary gap in primate default mode network organization. *Cell Rep.* **39**, 110669 (2022). 35417698 10.1016/j.celrep.2022.110669 PMC9088817

[14] Jing, W. et al. State-independent and state-dependent patterns in the rat default mode network. *Neuroimage* **237**, 118148 (2021). 33984491 10.1016/j.neuroimage.2021.118148

[15] Fox, K. C. R., Foster, B. L., Kucyi, A., Daitch, A. L. & Parvizi, J. Intracranial electrophysiology of the human default network. *Trends Cogn. Sci.*10.1016/j.tics.2018.02.002 (2018). 10.1016/j.tics.2018.02.002 PMC5957519 29525387

[16] Popa, D., Popescu, A. T. & Paré, D. Contrasting activity profile of two distributed cortical networks as a function of attentional demands. *J. Neurosci.* **29**, 1191–1201 (2009). 19176827 10.1523/JNEUROSCI.4867-08.2009 PMC2667329

[17] Hayden, B. Y., Smith, D. V. & Platt, M. L. Electrophysiological correlates of default-mode processing in macaque posterior cingulate cortex. *Proc. Natl. Acad. Sci. USA* **106**, 5948–5953 (2009). 19293382 10.1073/pnas.0812035106 PMC2667004

[18] Pais-Roldán, P. et al. Contribution of animal models toward understanding resting state functional connectivity. *Neuroimage* **245**, 118630 (2021). 34644593 10.1016/j.neuroimage.2021.118630 PMC9031339

[19] Nair, J. et al. Basal forebrain contributes to default mode network regulation. *Proc. Natl. Acad. Sci. USA* **115**, 1352–1357 (2018). 29363595 10.1073/pnas.1712431115 PMC5819396

[20] Nair, J. et al. Gamma band directional interactions between basal forebrain and visual cortex during wake and sleep states. *J. Physiol.* **110**, 19–28 (2016). 10.1016/j.jphysparis.2016.11.011 27913167

[21] Markello, R. D., Spreng, R. N., Luh, W. M., Anderson, A. K. & De Rosa, E. Segregation of the human basal forebrain using resting state functional MRI. *Neuroimage* **173**, 287–297 (2018). 29496614 10.1016/j.neuroimage.2018.02.042

[22] Li, J. et al. Mapping the subcortical connectivity of the human default mode network. *Neuroimage* **245**, 118758 (2021). 34838949 10.1016/j.neuroimage.2021.118758 PMC8945548

[23] Aguilar, D. D. & McNally, J. M. Subcortical control of the default mode network: Role of the basal forebrain and implications for neuropsychiatric disorders. *Brain Res. Bull.*10.1016/j.brainresbull.2022.05.005 (2022). 10.1016/j.brainresbull.2022.05.005 PMC9290753 35562013

[24] Alves, P. N. et al. An improved neuroanatomical model of the default-mode network reconciles previous neuroimaging and neuropathological findings. *Commun. Biol.* **2**, 370 (2019). 31633061 10.1038/s42003-019-0611-3 PMC6787009

[25] Harrison, B. J. et al. Dynamic subcortical modulators of human default mode network function. *Cereb. Cortex* **32**, 4345–4355 (2022). 34974620 10.1093/cercor/bhab487 PMC9528899

[26] Tu, W., Ma, Z., Ma, Y., Dopfel, D. & Zhang, N. Suppressing anterior cingulate cortex modulates default mode network and behavior in awake rats. *Cereb. Cortex* **31**, 312–323 (2021). 32820327 10.1093/cercor/bhaa227 PMC7727348

[27] Klaassen, A.-L., Heiniger, A., Vaca Sánchez, P., Harvey, M. A. & Rainer, G. Ventral pallidum regulates the default mode network, controlling transitions between internally and externally guided behavior. *Proc. Natl. Acad. Sci. USA* **118**, e2103642118 (2021). 34462351 10.1073/pnas.2103642118 PMC8433530

[28] Fakhraei, L. et al. Electrophysiological correlates of rodent default-mode network suppression revealed by large-scale local field potential recordings. *Cereb. Cortex Commun.* **2**, tgab034 (2021). 34296178 10.1093/texcom/tgab034 PMC8166125

[29] Giguere, M. & Goldman‐Rakic, P. S. Mediodorsal nucleus: areal, laminar, and tangential distribution of afferents and efferents in the frontal lobe of rhesus monkeys. *J. Comp. Neurol.* **277**, 195–213 (1988). 2466057 10.1002/cne.902770204

[30] Klein, J. C. et al. Topography of connections between human prefrontal cortex and mediodorsal thalamus studied with diffusion tractography. *Neuroimage* **51**, 555–564 (2010). 20206702 10.1016/j.neuroimage.2010.02.062 PMC2877805

[31] Krettek, J. E. & Price, J. L. The cortical projections of the mediodorsal nucleus and adjacent thalamic nuclei in the rat. *J. Comp. Neurol.* **171**, 157–191 (1977). 64477 10.1002/cne.901710204

[32] Roslin Sapawi, R. & Divac, I. Connections of the mediodorsal nucleus of the thalamus in the tree shrew. I. Afferent connections. *Neurosci. Lett.* **7**, 183–189 (1978). 19605110 10.1016/0304-3940(78)90165-9

[33] Divac, I. & Passingham, R. E. Connections of the mediodorsal nucleus of the thalamus in the tree shrew. II. Efferent connections. *Neurosci. Lett.* **19**, 21–26 (1980). 7052510 10.1016/0304-3940(80)90249-9

[34] Vives, F. & Mogenson, G. J. Electrophysiological evidence that the mediodorsal nucleus of the thalamus is a relay between the ventral pallidum and the medial prefrontal cortex in the rat. *Brain Res.* **344**, 329–337 (1985). 4041880 10.1016/0006-8993(85)90811-X

[35] Lavín, A. & Grace, A. A. Modulation of dorsal thalamic cell activity by the ventral pallidum: Its role in the regulation of thalamocortical activity by the basal ganglia. *Synapse* **18**, 104–127 (1994). 7839311 10.1002/syn.890180205

[36] Mogenson, G. J., Ciriello, J., Garland, J. & Wu, M. Ventral pallidum projections to mediodorsal nucleus of the thalamus: an anatomical and electrophysiological investigation in the rat. *Brain Res.* **404**, 221–230 (1987). 3032332 10.1016/0006-8993(87)91373-4

[37] Mitchell, A. S. The mediodorsal thalamus as a higher order thalamic relay nucleus important for learning and decision-making. *Neurosci. Biobehav. Rev.*10.1016/j.neubiorev.2015.03.001 (2015). 10.1016/j.neubiorev.2015.03.001 25757689

[38] Golden, E. C., Graff-Radford, J., Jones, D. T. & Benarroch, E. E. Mediodorsal nucleus and its multiple cognitive functions. *Neurology* **87**, 2161–2168 (2016). 27770073 10.1212/WNL.0000000000003344

[39] Preti, M. G., Bolton, T. A. & Van De Ville, D. The dynamic functional connectome: state-of-the-art and perspectives. *Neuroimage* **160**, 41–54 (2017). 28034766 10.1016/j.neuroimage.2016.12.061

[40] Yao, Y. G. Creating animal models, why not use the Chinese tree shrew (*Tupaia belangeri* chinensis)? *Zool. Res.* **38**, 118–126 (2017). 28585435 10.24272/j.issn.2095-8137.2017.032 PMC5460080

[41] Veit, J., Bhattacharyya, A., Kretz, R. & Rainer, G. Neural response dynamics of spiking and local field potential activity depend on CRT monitor refresh rate in the tree shrew primary visual cortex. *J. Neurophysiol.* **106**, 2303–2313 (2011). 21849615 10.1152/jn.00388.2011

[42] Bhattacharyya, A., Veit, J., Kretz, R., Bondar, I. & Rainer, G. Basal forebrain activation controls contrast sensitivity in primary visual cortex. *BMC Neurosci.* **14**, 55 (2013). 23679191 10.1186/1471-2202-14-55 PMC3662585

[43] Bhattacharyya, A., Bießmann, F., Veit, J., Kretz, R. & Rainer, G. Functional and laminar dissociations between muscarinic and nicotinic cholinergic neuromodulation in the tree shrew primary visual cortex. *Eur. J. Neurosci.* **35**, 1270–1280 (2012). 22487086 10.1111/j.1460-9568.2012.08052.x

[44] Dimanico, M. M. et al. Aspects of tree shrew consolidated sleep structure resemble human sleep. *Commun. Biol.* **4**, 722 (2021). 34117351 10.1038/s42003-021-02234-7 PMC8196209

[45] Marks, M. et al. Deep-learning-based identification, tracking, pose estimation and behaviour classification of interacting primates and mice in complex environments. *Nat. Mach. Intell.* **4**, 331–340 (2022). 35465076 10.1038/s42256-022-00477-5 PMC7612650

[46] Mathis, A. et al. DeepLabCut: markerless pose estimation of user-defined body parts with deep learning. *Nat. Neurosci.* **21**, 1281–1289 (2018). 30127430 10.1038/s41593-018-0209-y

[47] Wang, J., Karbasi, P., Wang, L. & Meeks, J. P. A layered, hybrid machine learning analytic workflow for mouse risk assessment behavior. *eNeuro* **10**, ENEURO.0335-22.2022 (2023). 36564214 10.1523/ENEURO.0335-22.2022 PMC9833056

[48] Ho, H. et al. A fully automated home cage for long-term continuous phenotyping of mouse cognition and behavior. *Cell Rep. Methods* **3**, 100532 (2023). 37533650 10.1016/j.crmeth.2023.100532 PMC10391580

[49] Luxem, K. et al. Open-source tools for behavioral video analysis: setup, methods, and best practices. *eLife* **12**, e79305 (2023). 36951911 10.7554/eLife.79305 PMC10036114

[50] Ide, K. & Takahashi, S. A review of neurologgers for extracellular recording of neuronal activity in the brain of freely behaving wild animals. *Micromachines*10.3390/mi13091529 (2022). 10.3390/mi13091529 PMC9502354 36144152

[51] Berens, P. CircStat: a MATLAB toolbox for circular statistics. *J. Stat. Softw*. **31**, 1–21 (2009).

[52] Malhotra, S., Cross, R. W., Zhang, A. & Van Der Meer, M. A. A. Ventral striatal gamma oscillations are highly variable from trial to trial, and are dominated by behavioural state, and only weakly influenced by outcome value. *Eur. J. Neurosci.* **42**, 2818–2832 (2015). 26363137 10.1111/ejn.13069

[53] Heimer, L., Switzer, R. D. & Van Hoesen, G. W. Ventral striatum and ventral pallidum. Components of the motor system? *Trends Neurosci.*10.1016/0166-2236(82)90037-6 (1982).

[54] Lozano-Montes, L. et al. Optogenetic stimulation of basal forebrain parvalbumin neurons activates the default mode network and associated behaviors. *Cell Rep.* **33**, 108359 (2020). 33176133 10.1016/j.celrep.2020.108359

[55] Stratford, T. R. & Wirtshafter, D. Evidence that the nucleus accumbens shell, ventral pallidum, and lateral hypothalamus are components of a lateralized feeding circuit. *Behav. Brain Res.* **226**, 548–554 (2012). 22019344 10.1016/j.bbr.2011.10.014 PMC3251216

[56] Stratford, T. R., Kelley, A. E. & Simansky, K. J. Blockade of GABA(A) receptors in the medial ventral pallidum elicits feeding in satiated rats. *Brain Res.* **825**, 199–203 (1999). 10216189 10.1016/S0006-8993(99)01239-1

[57] Dwiel, L. L., Khokhar, J. Y., Connerney, M. A., Green, A. I. & Doucette, W. T. Finding the balance between model complexity and performance: using ventral striatal oscillations to classify feeding behavior in rats. *PLoS Comput. Biol.* **15**, e1006838 (2019). 31009448 10.1371/journal.pcbi.1006838 PMC6497302

[58] Zimmerman, C. A., Leib, D. E. & Knight, Z. A. Neural circuits underlying thirst and fluid homeostasis. *Nat. Rev. Neurosci.*10.1038/nrn.2017.71 (2017). 10.1038/nrn.2017.71 PMC5955721 28638120

[59] Abbott, S. B. G., Machado, N. L. S., Geerling, J. C. & Saper, C. B. Reciprocal control of drinking behavior by median preoptic neurons in mice. *J. Neurosci.* **36**, 8228–8237 (2016). 27488641 10.1523/JNEUROSCI.1244-16.2016 PMC4971367

[60] Chen, X. et al. Resting-state functional network connectivity underlying eating disorder symptoms in healthy young adults. *Neuroimage Clin.* **30**, 102671 (2021). 33892431 10.1016/j.nicl.2021.102671 PMC8082688

[61] McFadden, K., Tregellas, J., Shott, M. & Frank, G. Reduced salience and default mode network activity in women with anorexia nervosa. *J. Psychiatry Neurosci.* **39**, 178–188 (2014). 24280181 10.1503/jpn.130046 PMC3997603

[62] Kalueff, A. V. et al. Neurobiology of rodent self-grooming and its value for translational neuroscience. *Nat. Rev. Neurosci.*10.1038/nrn.2015.8 (2016). 10.1038/nrn.2015.8 PMC4840777 26675822

[63] Fang, H. et al. High activity of the stress promoter contributes to susceptibility to stress in the tree shrew. *Sci. Rep.* **6**, 24905 (2016). 27125313 10.1038/srep24905 PMC4850381

[64] Zeev-Wolf, M., Levy, J., Goldstein, A., Zagoory-Sharon, O. & Feldman, R. Chronic early stress impairs default mode network connectivity in preadolescents and their mothers. *Biol. Psychiatry Cogn. Neurosci. Neuroimaging* **4**, 72–80 (2019). 30446436 10.1016/j.bpsc.2018.09.009

[65] Li, G. et al. Neural correlates of posttraumatic anhedonia symptoms: decreased functional connectivity between ventral pallidum and default mode network regions. *J. Psychiatr. Res.* **140**, 30–34 (2021). 34090100 10.1016/j.jpsychires.2021.05.061

[66] Caldji, C. et al. Maternal care during infancy regulates the development of neural systems mediating the expression of fearfulness in the rat. *Proc. Natl. Acad. Sci. USA* **95**, 5335–5340 (1998). 9560276 10.1073/pnas.95.9.5335 PMC20261

[67] Martin, R. D. Tree shrews: unique reproductive mechanism of systematic importance. *Science* **152**, 1402–1404 (1966). 5937138 10.1126/science.152.3727.1402

[68] Staudigl, T. et al. Memory signals from the thalamus: early thalamocortical phase synchronization entrains gamma oscillations during long-term memory retrieval. *Neuropsychologia* **50**, 3519–3527 (2012). 22975190 10.1016/j.neuropsychologia.2012.08.023

[69] Buckner, R. L., Andrews‐Hanna, J. R. & Schacter, D. L. The brain’s default network. *Ann. N. Y Acad. Sci.* **1124**, 1–38 (2008). 18400922 10.1196/annals.1440.011

[70] Greicius, M. D., Krasnow, B., Reiss, A. L. & Menon, V. Functional connectivity in the resting brain: a network analysis of the default mode hypothesis. *Proc. Natl. Acad. Sci. USA* **100**, 253–258 (2003). 12506194 10.1073/pnas.0135058100 PMC140943

[71] Divac, I. & Passingham, R. E. Connections of the mediodorsal nucleus of the thalamus in the tree shrew. II Efferent connections. *Neurosci. Lett.* **19**, 21–26 (1980). 7052510 10.1016/0304-3940(80)90249-9

[72] Root, D. H., Melendez, R. I., Zaborszky, L. & Napier, T. C. The ventral pallidum: subregion-specific functional anatomy and roles in motivated behaviors. *Progr. Neurobiol.*10.1016/j.pneurobio.2015.03.005 (2015). 10.1016/j.pneurobio.2015.03.005 PMC4687907 25857550

[73] Ouhaz, Z., Fleming, H. & Mitchell, A. S. Cognitive functions and neurodevelopmental disorders involving the prefrontal cortex and mediodorsal thalamus. *Front. Neurosci.*10.3389/fnins.2018.00033 (2018). 10.3389/fnins.2018.00033 PMC5808198 29467603

[74] Alderson, T. et al. Disrupted thalamus white matter anatomy and posterior default mode network effective connectivity in amnestic mild cognitive impairment. *Front. Aging Neurosci.* **9**, 370 (2017). 29167639 10.3389/fnagi.2017.00370 PMC5682321

[75] Shine, J. M., Lewis, L. D., Garrett, D. D. & Hwang, K. The impact of the human thalamus on brain-wide information processing. *Nat. Rev. Neurosci.* **24**, 416–430 (2023). 37237103 10.1038/s41583-023-00701-0 PMC10970713

[76] Shine, J. M. et al. The low-dimensional neural architecture of cognitive complexity is related to activity in medial thalamic nuclei. *Neuron* **104**, 849–855.e3 (2019). 31653463 10.1016/j.neuron.2019.09.002

[77] Furth, K. E. et al. Neuronal correlates of ketamine and walking induced gamma oscillations in the medial prefrontal cortex and mediodorsal thalamus. *PLoS ONE* **12**, e0186732 (2017). 29095852 10.1371/journal.pone.0186732 PMC5667758

[78] Pergola, G. et al. The regulatory role of the human mediodorsal thalamus. *Trends Cogn. Sci.*10.1016/j.tics.2018.08.006 (2018). 10.1016/j.tics.2018.08.006 PMC6198112 30236489

[79] Ray, S. & Maunsell, J. H. R. Different origins of gamma rhythm and high-gamma activity in macaque visual cortex. *PLoS Biol.* **9**, e1000610 (2011). 21532743 10.1371/journal.pbio.1000610 PMC3075230

[80] Ray, S., Crone, N. E., Niebur, E., Franaszczuk, P. J. & Hsiao, S. S. Neural correlates of high-gamma oscillations (60-200 Hz) in macaque local field potentials and their potential implications in electrocorticography. *J. Neurosci.* **28**, 11526–11536 (2008). 18987189 10.1523/JNEUROSCI.2848-08.2008 PMC2715840

[81] Emmons, L. H. *Tupai* (University of California Press, 2000).

[82] López-Azcárate, J. et al. Delta-mediated cross-frequency coupling organizes oscillatory activity across the rat cortico-basal ganglia network. *Front. Neural Circuits* **7**, 155 (2013). 24106462 10.3389/fncir.2013.00155 PMC3788338

[83] Smith, K. Lab mice go wild: making experiments more natural in order to decode the brain. *Nature*10.1038/d41586-023-01926-w (2023). 10.1038/d41586-023-02303-3 37524963

[84] Nath, T. et al. Using DeepLabCut for 3D markerless pose estimation across species and behaviors. *Nat. Protoc.* **14**, 2152–2176 (2019). 31227823 10.1038/s41596-019-0176-0

[85] Conners, M. G. et al. Hidden Markov models identify major movement modes in accelerometer and magnetometer data from four albatross species. *Mov. Ecol.* **9**, 7 (2021). 33618773 10.1186/s40462-021-00243-z PMC7901071

[86] Kendall-Bar, J. M. et al. Brain activity of diving seals reveals short sleep cycles at depth. *Science* **380**, 260–265 (2023). 37079694 10.1126/science.adf0566

[87] Barnett, L. & Seth, A. K. The MVGC multivariate Granger causality toolbox: a new approach to Granger-causal inference. *J. Neurosci. Methods* **223**, 50–68 (2014). 24200508 10.1016/j.jneumeth.2013.10.018

