# Author Correction: Mediodorsal thalamus and ventral pallidum contribute to subcortical regulation of the default mode network

**DOI:** 10.1038/s42003-024-06651-2

**Published:** 2024-08-08

**Authors:** Yilei Zhao, Tobias Kirschenhofer, Michael Harvey, Gregor Rainer

**Affiliations:** Section of Medicine, Faculty of Science and Medicine, University of Fribourg, Fribourg, Switzerland

**Keywords:** Neural circuits, Cognitive control

Correction to: *Communications Biology* 10.1038/s42003-024-06531-9, published online 23 July 2024

In this article the grant number 182504 relating to Swiss National Science Foundation for G. Rainer was omitted. The original article has been corrected.

